# Signal Transduction as an Assimilation of Signals with Different Origins and Different Intracellular States

**DOI:** 10.3390/ijms241210085

**Published:** 2023-06-13

**Authors:** Klaus Holzmann, Hedwig Sutterlüty

**Affiliations:** Center for Cancer Research, Comprehensive Cancer Center, Medical University Vienna, Borschkegasse 8a, 1090 Vienna, Austria

Higher organisms, such as humans, are made up of trillions of cells that have to act as a unit in a finely tuned way to ensure the functioning of the living being that is composed of them. The delicate coordination of vital processes requires sophisticated communication. This takes place when cells emit signals with different ranges and the totality of the cellular environment acts on the cells [1]. Depending on the intracellular composition, specific interpretations cause cells to react in a certain way to this cocktail of signals.

The receiving cell recognizes signals via specific receptors, and a precisely coordinated network of transmitting, attenuating, and enhancing molecules ensures that the transmission is precisely tuned in terms of time and space, such that a specific cellular response is guaranteed. Important biological processes, such as cell proliferation, migration, and growth on the one hand with differentiation as well as cell death on the other, are largely regulated in this way. It is therefore not surprising that, in cancer, this finely orchestrated regulatory system is affected by mutations, deletions, and amplifications [2]. Resulting malfunctions and expression levels are the reasons why cells no longer carry out these processes in coordination with cell association.

In this Special Issue, several publications dealing with signal transduction in normal and tumor cells were collected. Four original works and one review article focus on the molecules or mechanisms involved in the transduction of signals.

Although signals are mostly of a chemical nature, physical forces can also function as signals that induce a cellular response, decisively influencing the fate of a cell. In this context, Dadwal et al. [3], investigate to what extent three-dimensional scaffolds with different pore sizes influence biological processes in tumor cell lines isolated as bone metastases. The data demonstrate that these structural features influence important biological processes, such as proliferation and migration. The altered cell behavior can be explained via the observation of specific differences in the gene expression profile. In particular, molecules known to frequently change their expression in bones affected by tumors are more strongly expressed in the tumor cells grown in these three-dimensional structures. Remarkably, it was noted that the rigidity and pore size of the 3D scaffolds additionally influenced the sensitivity of the cells to therapeutic agents, such as inhibitors of integrin, TGFbeta, and Gli. In mice, the infiltration of xenografts by macrophages is also affected by these structures. These data demonstrate that cells integrate chemical and structural signals in a cellular response. Such multifaceted 3D models can simulate the morphometric properties of a long bone and may be beneficial for investigations of questions that include tumor-induced bone diseases.

Puckett et al. [4] enrich this Special Issue with a very comprehensive review where the authors describe the role of pyruvate kinase M2 (PKM2) in normal and tumor cells. This enzyme is one of four different isoforms and is produced as a splice form of the PKM gene locus. Pyruvate kinase catalyzes one of many reactions in glycolysis, namely that which converts phosphoenolpyruvate and ADP into pyruvate and ATP. Compared to the other isoforms, PKM2 is less active and participates primarily in embryonic developmental processes. In adults, higher expression is found mainly in tumors but also in other diseases. In this review, the multiple mechanisms regulating the expression of this molecule are comprehensively described. A major focus is placed on those observations that involve non-coding RNAs as regulators of PKM2. Work on microRNAs, as well as long non-coding RNAs and circular RNAs, is listed and broadly discussed. In addition to expression, PKM2 activity and localization are regulated by mechanisms that can integrate many signaling pathways. This review goes on to describe how PKM2 can be translocated to the nucleus, where it can function as a protein kinase to affect the gene expression of important molecules, such as MXY and CCND1. In addition, the authors address mutations in this enzyme found in cancers and discuss how these mutations may affect the activity of the protein, nicely outlining how PKM2 may well have a regulatory role in addition to its enzymatic function in the frequently observed metabolic transformation of the tumor cell. This review article integrates PKM2 into a network in which external factors, such as food supply, but also oxidative stress and radiation, are processed into a cellular reaction.

The remaining three reports deal with molecules that regulate signal transduction within a cell:

Salvi et al. [5] studied the influence of glycosylation on the proteome of cells and discovered that signaling induced by insulin-like growth factor 1 is especially vulnerable to defects in steps of this modification procedure. Membrane proteins, including receptors, are particularly frequently glycosylated, and through this post-translational modification the structural properties of the molecules are influenced. The proteome of parental CHO cells was compared with that of two cell lines lacking either alpha-1,6-mannosylglycoprotein 6-beta-N-acetylglucosaminyltransferase 1 (MGAT1) or MGAT5. Both enzymes are essential for the N-glycosylation of proteins. This analysis showed that over 40 membrane proteins were strongly differentially expressed in both glycosylation-defective cell lines. It was shown that, in addition to a greatly increased presence of integrins in the cell membrane, proteins that can be assigned to the network regulating cellular signaling were commonly affected by the absence of this modification. In particular, the insulin-like growth factor 1 receptor (IGF-1R) and the IQ motif-containing GTPase-activating protein (IQGAP1) were strongly repressed in both cell lines. As a result, IGF-1-induced signal transduction was also strongly reduced. This article demonstrates that the influence of a post-translational modification can determine the response of cells toward extracellular cues.

Finally, two original articles report how signaling can be influenced by different isoforms of Sprouty proteins. Sprouty proteins are modulators of receptor-tyrosine-kinase-induced signal transmission. The work of Kamptner et al. investigates the function of Sprouty1 and Sprouty3 in osteosarcoma-derived cells [6]. The experiments described therein show that Sprouty1 has no significant effect on the biological processes involved in malignancies such as proliferation, migration, or spheroid formation, whereas the ectopic expression of Sprouty3 promotes all three processes. A dominant-negative version of Sprouty3 interferes with cell migration. Corroborating this, protein levels of Sprouty3 are expressed to a greater extent in malignant bone cells as compared to normal primary fibroblasts. This study shows that Sprouty3 fulfills tumor-promoting functions in osteosarcoma. It supplements an earlier study investigating the role of Sprouty2 and Sprouty4 in the same tumor entity [7], and together these papers demonstrate that the different Sprouty isoforms administrate different regulatory roles in the cancerogenesis of osteosarcoma.

A genetic variation of Sprouty4 is investigated in the original article of Stütz et al. [8]. It was earlier identified in patients with Kallmann syndrome, which is a hereditary disease that is mostly associated with a reduced effect of FGF-induced signaling. The mutations mainly associated with the syndrome involve the anosmin1 and the FGFR1 receptor genes. Very rarely, mutations are also found in the *Sprouty4 gene* [9,10,11,12]. The most common of these Spry4 mutations in Kallmann syndrome patients was studied in this article. The results show that this mutation generates a hyperactive form of Spry4 in fibroblasts. In contrast to the unmodified protein, the variant is able to inhibit the migration and proliferation of primary lung fibroblasts. This is likely due to the inhibition of the MAPK pathway. This hyperactivity is also observed in osteosarcoma-derived cells. The data in this article demonstrate that an alteration in Sprouty4 influences important cellular processes in fibroblasts and suggest a mode of action as to how this mutation may contribute to the phenotype of Kallmann syndrome patients.

In summary, this Special Issue deals with different aspects of intercellular communication through signal processing. On the one hand, it is shown that different signals can be recognized by recipient cells and integrated into a cellular response. On the other hand, recent data are presented that show how intracellular changes can modulate the processing of signals, thus altering their effect. The publications presented here contribute to the understanding of the multifaceted aspects of signal transduction and promote the further development of research opportunities for the targeted therapy of cancer.
